# Constructing Prediction Models for Freezing of Gait by Nomogram and Machine Learning: A Longitudinal Study

**DOI:** 10.3389/fneur.2021.684044

**Published:** 2021-12-06

**Authors:** Kun Xu, Xiao-xia Zhou, Run-cheng He, Zhou Zhou, Zhen-hua Liu, Qian Xu, Qi-ying Sun, Xin-xiang Yan, Xin-yin Wu, Ji-feng Guo, Bei-sha Tang

**Affiliations:** ^1^Collaborative Innovation Center for Brain Disorders, Laboratory of Brain Disorders, Ministry of Science and Technology, Beijing Institute of Brain Disorders, Capital Medical University, Beijing, China; ^2^Department of Neurology, Xiangya Hospital, Central South University, Changsha, China; ^3^Department of Geriatrics, Xiangya Hospital, Central South University, Changsha, China; ^4^Department of Epidemiology and Health Statistics, Xiangya School of Public Health, Central South University, Changsha, China; ^5^National Clinical Research Center for Geriatric Disorders, Xiangya Hospital, Central South University, Changsha, China; ^6^Centre for Medical Genetics, Hunan Key Laboratory of Medical Genetics, School of Life Sciences, Central South University, Changsha, China; ^7^Key Laboratory of Hunan Province in Neurodegenerative Disorders, Central South University, Changsha, China

**Keywords:** freezing of gait, risk factors, prediction model, statistics, machine learning

## Abstract

**Objectives:** Although risk factors for freezing of gait (FOG) have been reported, there are still few prediction models based on cohorts that predict FOG. This 1-year longitudinal study was aimed to identify the clinical measurements closely linked with FOG in Chinese patients with Parkinson's disease (PD) and construct prediction models based on those clinical measurements using Cox regression and machine learning.

**Methods:** The study enrolled 967 PD patients without FOG in the Hoehn and Yahr (H&Y) stage 1–3 at baseline. The development of FOG during follow-up was the end-point. Neurologists trained in movement disorders collected information from the patients on a PD medication regimen and their clinical characteristics. The cohort was assessed on the same clinical scales, and the baseline characteristics were recorded and compared. After the patients were divided into the training set and test set by the stratified random sampling method, prediction models were constructed using Cox regression and random forests (RF).

**Results:** At the end of the study, 26.4% (255/967) of the patients suffered from FOG. Patients with FOG had significantly longer disease duration, greater age at baseline and H&Y stage, lower proportion in Tremor Dominant (TD) subtype, a higher proportion in wearing-off, levodopa equivalent daily dosage (LEDD), usage of L-Dopa and catechol-O-methyltransferase (COMT) inhibitors, a higher score in scales of Unified Parkinson's Disease Rate Scale (UPDRS), 39-item Parkinson's Disease Questionnaire (PDQ-39), Non-Motor Symptoms Scale (NMSS), Hamilton Depression Rating Scale (HDRS)-17, Parkinson's Fatigue Scale (PFS), rapid eye movement sleep behavior disorder questionnaire-Hong Kong (RBDQ-HK), Epworth Sleepiness Scale (ESS), and a lower score in scales of Parkinson's Disease Sleep Scale (PDSS) (*P* < 0.05). The risk factors associated with FOG included PD onset not being under the age of 50 years, a lower degree of tremor symptom, impaired activities of daily living (ADL), UPDRS item 30 posture instability, unexplained weight loss, and a higher degree of fatigue. The concordance index (C-index) was 0.68 for the training set (for internal validation) and 0.71 for the test set (for external validation) of the nomogram prediction model, which showed a good predictive ability for patients in different survival times. The RF model also performed well, the C-index was 0.74 for the test set, and the AUC was 0.74.

**Conclusions:** The study found some new risk factors associated with the FOG including a lower degree of tremor symptom, unexplained weight loss, and a higher degree of fatigue through a longitudinal study, and constructed relatively acceptable prediction models.

## Introduction

Parkinson's disease (PD) is the most common type of Parkinsonism with a complex spectrum of motor and non-motor characteristics (1). Some patients with PD may suffer freezing of gait (FOG), a common motor complication of PD, which occurs in different stages of PD especially common in the advanced stage and when the drug efficacy wanes (wearing off). FOG is a sudden, variable, and often unpredictable transient break in walking, always occurring at the beginning of or during gait, especially while turning, leading to falls. Patients report that FOG feels like their feet are stuck to the floor (2–4). The Unified Parkinson's Disease Rate Scale (UPDRS) scale is often used in assessing gait abnormalities (5). FOG occurs not only in advanced PD but also in other parkinsonism, especially in progressive supranuclear palsy (PSP), multiple system atrophy (MSA), corticobasal degeneration (CBD), dementia with Lewy bodies (DLB) (6). The prevalence of FOG in patients with PD varies from 14.0 to 55.1%, and in patients with advanced PD, up to 86.5% suffer from FOG (7, 8). The prevalence of FOG in PSP is about 53%, 54% in MSA, 54% in DLB, and 25% in CBD, and is commonly seen in the late stages of these diseases (6).

Freezing of gait (FOG) significantly affects the quality of life (QoL) of patients with PD, and due to the clinical heterogeneity of FOG, the effect of treatment that is mainly based on drug and non-drug therapies is not ideal, especially when Dopa-responsive FOG turns into Dopa-resistance FOG (9–11). Previous studies have explored risk factors and biomarkers and constructed prediction models of certain neurodegenerations (12). In addition, identifying risk factors is also conducive to finding out new therapeutic targets of neurodegenerations (13, 14).

Many risk factors for FOG also have been reported by previous cross-sectional clinical studies and some longitudinal studies (11), however, cross-sectional clinical studies cannot completely describe the development and progression of FOG and there are still few prediction models based on cohorts that predict FOG. One model with different risk factors included caudate Dopamine Transporter (DAT) uptake and Cerebro-Spinal Fluid (CSF) Aβ_42_ that showed a good predictive ability (15). However, because the examination of caudate DAT uptake is complex and expensive, and the examination of CSF Aβ42 is invasive and has certain risks, it is not suitable for large-scale clinical screening and promotion. Another prediction model constructed by a prospective multimodal neuroimaging cohort study showed accuracy, finding that thalamic gray matter inflation was a predictor of FOG (16). Some other studies used clinical assessment scales to identify risk factors of diseases (17). A longitudinal study proved that prediction models that were constructed by clinical scales could show good prediction ability (18).

Machine learning (ML) uses statistical and mathematical algorithms to develop models that can predict an outcome based on factors related to that outcome. Prediction models from machine learning processes are used in medical fields to diagnose and assess disease progression and evaluate prognosis, which strengthened personalized healthcare and improved strategies for disease treatment (19).

This prospective study used different clinical rating scales in the Parkinson's Disease and Movement Disorders Multicenter Database and Collaborative Network in China (PD-MDCNC; http://www.pd-mdcnc.com/), and the present prospective study was aimed to find out some clinical characteristics in FOG patients, identify some risk factors of FOG, and construct prediction models of FOG.

## Materials and Methods

**Figure 1:**
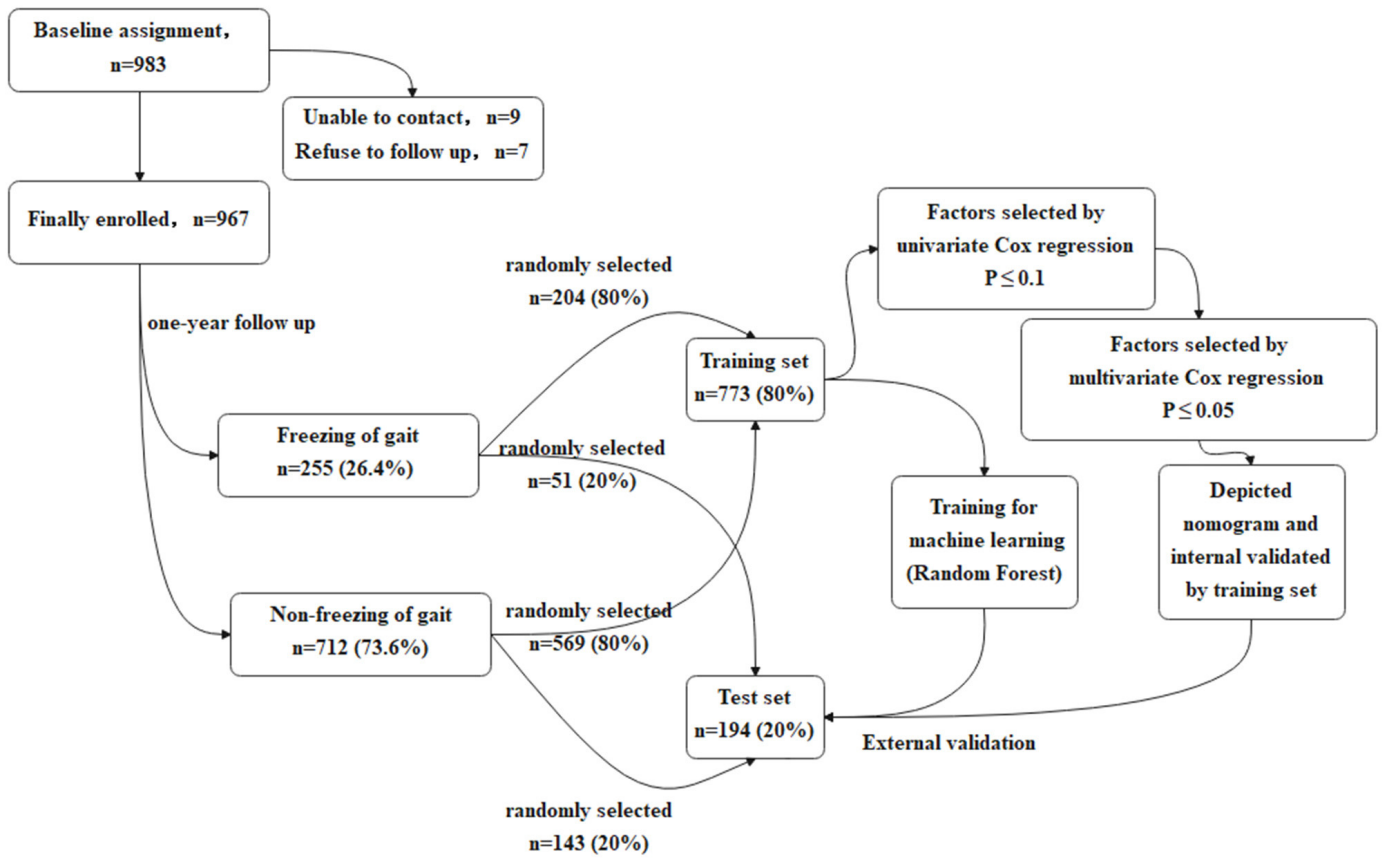
The flow chart of the study.

In order to make the study procedures more clear and easy to understand, the flow chart of the study is presented in Figure 1.

### Study Population

Patients with PD were recruited from the Department of Neurology of Xiangya Hospital Central South University in China from January 1, 2017 to July 31, 2019. All the patient baseline health and disease characteristics including the clinical scales scores were entered into the PD-MDCNC database.

The inclusion criteria included: (a) PD diagnosis (20); (b) available clinical data; (c) at least 1 year of disease duration and at least 1 year of a history of anti-PD drug therapy; (d) non-FOG at baseline; (e) non-L-Dopa-induced dyskinesia; (f) Hoehn and Yahr (H&Y) stage 1–3; (g) agreement for a 1-year follow-up.

The exclusion criteria included: (a) prior history of neuropsychiatric disorders (Alzheimer's disease, schizophrenia) before PD diagnosis; (b) history of diseases that may be confused with the non-motor symptoms of PD (allergic rhinitis, prostatitis); (c) refused follow-up; (d) patients with a disability causing gait disorder.

Both baseline and the first follow-up assessment that is carried out after 1 year were implemented in Xiangya Hospital. The interval of follow-up for each patient was 1 year. Patients with a medication history are more likely to develop FOG (11). In our study, to explore the impact of the baseline drug consumption on FOG, we recruited patients with PD with at least 1 year of disease duration and at least 1 year of a history of anti-PD drug therapy. The survival time of the patients with FOG in Cox regression was set as “disease duration at baseline plus 1 year”. Whenever the patients began experiencing FOG during the follow-up, they were recorded as “End with FOG.” What is more, due to the New Freezing of Gait-Questionnaire (NFOG-Q), a freezer was identified only when FOG-episodes were present during the past month, suggesting that the time frame was short enough for recall and long enough for identifying a consistent pattern (21).

There were 983 patients with PD being assessed at the baseline, and during the 1-year follow-up, 16 patients quit. Finally, 967 patients with PD (496 men and 471 women) were enrolled in the study. Each patient in the study provided written informed consent. The study was reviewed and approved by the Ethics Committee of Xiangya Hospital of Central South University in China.

### Variables

For the statistical calculations, prediction model development, and ML, the characteristics of the patients with PD were identified as “variables.” For example, PD medication regimen and clinical characteristics such as different motor and non-motor symptoms were called “independent variables,” and the situation that patients end with FOG was called the outcome.

#### Outcomes

The UPDRS-14 (22) and the NFOG-Q (21) were used to determine the outcome “End with FOG,” which was defined as “UPDRS-14 score ≥1” and “NFOG-Q evaluation of FOG” (NFOG-Q score also >1). Meanwhile, the experts would provide a detailed description of FOG that “Have you ever feel that your feet are glued to the ground when you walk, turn, or intent to start a walking during the recent months?” to every patient. To those patients who did not determine whether they suffered FOG, experts would imitate all of the subtypes of FOG to ensure the accuracy of the data. Combined with the answer of the patients to the scales and the intervention of experts, the outcome, “End with FOG,” would be finally determined. Patients diagnosed with FOG were placed into a FOG (+) category. At the end of the study, the patients at follow-up that were not diagnosed with FOG were placed into an FOG (–) category. All patients were assessed in the “OFF” medication state. In this study, the “OFF” medication state was defined as: PD patients did not take the medicine at outpatient service, or after taking the medicine they had no efficacy or were in a relatively poor state throughout the day.

#### Independent Variables and Clinical Assessments

The independent variables in this study were derived from the clinical rating scales in the PD-MDCNC database, each of which had strict scientific significance. Baseline characteristics were collected from the subjects by neurologists trained in movement disorders. The characteristics included sex, age, education level, smoking history, history of alcohol intake, history of exposure to toxic and hazardous substances (including pesticides and organic solvents), history of exposure to heavy metal, history of head injury, surgical history, family history of PD, and age of onset (AOO), duration. The PD medication regimen included L-Dopa, catechol-O-methyltransferase (COMT) Inhibitors, anticholinergic drugs, amantadine, dopamine agonists, and MAO-B inhibitors, and total levodopa equivalent daily dosage (LEDD) (mg/day) (23).

Age at baseline was divided into five categories: 1 means < 50 years old, 2 means age at baseline ≥ 50 and ≤ 59, 3 means age at baseline ≥ 60 and ≤ 69, and similarly so, up to 5 means > 80 years old. In fact, in the process of constructing the models, we tried to input the age of every single patient into the models, while after several experiments of input method, we chose to divide the patients into different age groups, which was the most appropriate classification method for constructing prediction models based on this cohort data. To group AOO, early-onset PD (EOPD) (+) was defined as AOO ≤ 50 years old and EOPD (–) was defined as AOO ≥ 50 years old (24), and factors of AOO were named as EOPD (±).

Motor symptoms were evaluated using the separate scores of each item of UPDRS and total score of parts I, II (excluding the score from item 14), III, and the whole UPDRS, as well as the H&Y stage (1–3). The tremor dominant (TD) and postural instability gait difficulty (PIGD) scores were determined to classify patients as TD, PIGD, and “intermediate” (25). The 39-item Parkinson's Disease Questionnaire (PDQ-39) was used to evaluate the overall situation of patients (26). The Non-Motor Symptoms Scale (NMSS) (27) was used to evaluate the global non-motor symptoms. The mini-mental state examination (MMSE) (28) was used to evaluate the cognitive function of PD patients. The Hamilton Depression Rating Scale (HDRS-17) (29) was used to assess depression. Rome III criteria (IBS-C) were used to diagnose functional constipation (30). The Parkinson's Fatigue Scale (PFS-16) (31) and Epworth Sleepiness Scale (ESS) (32) were used to evaluate fatigue and excessive daytime sleepiness (EDS). The Hyposmia Rating Scale (HRS) (33) and the rapid eye movement (REM) sleep behavior disorder questionnaire-Hong Kong (RBDQ-HK) (34) were used to evaluate hyposmia and REM sleep behavior disorder (RBD).

All of the above-mentioned assessments were conducted in the “OFF” medication state.

### Statistical Analysis

#### Sample Characteristics

Proportions (%) were used to describe unordered classified variables and means and SD (mean, SD) were used to describe continuous variables. Statistical differences between the FOG (+) and FOG (–) groups were determined with the Chi-square test, the Student's *t*-test, and the Mann-Whitney *U*-test using Statistical Package for the Social Sciences (SPSS) version 25.0 (IBM, Armonk, New York, United States). Statistical analyses were performed as two-tailed tests and the threshold of significance (α) was 0.05.

#### The Training Set and the Test Set

Stratified random sampling was used to divide the population into two independent sets: a training set and a test set. The training set included 80% (773/967) of the study cohort, randomly selected from the FOG+ (204/255) and FOG- (569/712) groups. Models were developed using the characteristics in the training set. The test set was used to validate the models, including the remaining 20% (194/967) of the study cohort. Partition of the data set was realized by the train_test_split function in the sklearn package Python 3.7.0. In the partitioning, random_state was set to 0.

#### Cox Regression

Cox regression was used to determine the hazard ratios (HRs) for variables contributing to FOG and to construct a model to predict FOG. A univariate Cox regression model was constructed for each variable to determine the statistical significance of the model and only those variables with *P* ≤ 0.1 were screened in the next stage of the analysis. Results with clinical significance were screened out and a multivariate Cox regression with a forward likelihood ratio (LR) model was performed to find risk factors for FOG with a threshold of *P* ≤ 0.05. The survival time was set as the disease duration at the baseline plus 1 year. Kaplan–Meier survival curves were constructed by the training set, and log-rank tests were performed to determine if significant differences existed in survival times between the FOG(+) and FOG(–) groups. Nomograms were constructed to transform the complex regression equations into visual graphs (RMS 6.1-0 in R 4.0.3; http://www.r-project.org/). To verify the model, using the training set for internal validation, and the test set for external validation, respectively. Bootstrap self-sampling was used for validation. Concordance index (C-index) and calibration curve were used to evaluate model fit to the data (RMS 6.1-0 in R 4.0.3; http://www.r-project.org/). Time-dependent Receiver Operating Characteristic Curves (tdROC) were depicted using survComp (R 4.0.3; http://www.r-project.org/). Areas under the curves (AUC) were used to assess the prediction accuracy of the model at various survival times for internal and external validation.

### Machine Learning

Random Forest (RF) was used to develop an alternative prediction model for FOG. The bootstrap self-sampling was used to randomly extract ~2/3 of the samples from the training set for the construction of each decision tree. Next, some factors were randomly extracted from all the features during the construction of each decision tree, and those variables with the best classification ability were selected for the construction of the tree. The importance of factors in the construction of decision trees was measured using Gini-index (https://data.worldbank.org/indicator/SI.POV.GINI). The RF model was constructed using the RF Classifier function in the sklearn package Python 3.7.0. Relevant parameters were adjusted using gridSearchCV in Python 3.7.0.

### Mutual Benefits

Cox regression, especially, is applied on the occasion when the outcome variables are closely related to the disease progression, so the Cox regression was chosen as the main analytical method of our study. RF is a common machine learning algorithm that is applied to classification and prediction, especially in regression and classification scenarios, indicating that RF is a way to combine and validate Cox regression. The importance of every single factor for FOG would be ranked by the Gini-index in RF. After the importance of different factors in the Gini-rank was compared, the factors found in the Cox regression could be validated. What is more, other machine learning approaches such as support vector machines (SVM), Random survival forest (RSF) did not achieve our desired results, suggesting that RF might be the best ML approach to this investigation of this dataset.

## Results

### Baseline Population Characteristics

All baseline features for the patients with FOG (–) and FOG (+) are presented in Tables 1–3.

**Table 1:** Baseline characteristics of demographic and drug consuming of the population.

	**Total** ***n* = 967**	**FOG (-)** ***n* = 712**	**FOG (+)** ***n* = 255**	**Test**	***P*-value**
Sex (Male, %)	496 (51.3)	377 (52.9)	119 (46.7)	2	0.093
Disease duration, mean (SD), y	5.93 (3.63)	5.48 (3.37)	7.18 (4.02)	1	0.000*
Age at baseline, mean (SD), y	62.61 (10.01)	61.89 (9.94)	64.62 (9.94)	1	0.000*
Age of onset, mean (SD), y	55.68 (10.28)	55.41 (10.21)	56.44 (10.44)	1	0.174
EOPD (%)	307 (31.7)	232 (32.6)	75 (29.4)	2	0.389
Family history of PD (%)	95 (9.8)	77 (10.8)	18 (7.1)	2	0.087
Education level, high (%)	150 (15.5)	117 (16.4)	33 (12.9)	2	0.226
Smoking history, current (%)	242 (25%)	184 (25.8%)	58 (22.7%)	2	0.355
Alcohol intake history, current (%)	215 (22.2)	165 (23.2)	50 (19.6)	2	0.255
History of exposure to pesticide (%)	69 (7.1)	52 (7.3)	17 (6.7)	2	0.887
History of exposure to organic solvent (%)	20 (2.1)	18 (2.5)	2 (0.8)	2	0.123
History of exposure to heavy metal contamination (%)	29 (3)	23 (3.2)	6 (2.4)	2	0.669
Head injury (%)	42 (4.3)	35 (4.9)	7 (2.7)	2	0.157
Operation history (%)	230 (23.8)	166 (23.3)	64 (25.1)	2	0.607
Age category (1–5)	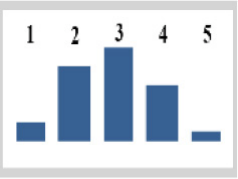	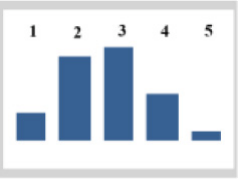	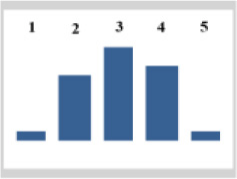	3	0.000*
**Drug consuming**					
LEDD	315.57 ± 174.97	299.62 ± 170.32	360.08 ± 180.35	1	0.000*
Wearing-off (yes, %)	125 (12.90%)	74 (10.39%)	51 (20.00%)	2	0.000*
Levo-Dopa (yes, %)	883 (91.3%)	635 (89.19%)	248 (97.25%)	2	0.000*
COMT inhibitors (yes, %)	44 (4.6%)	23 (3.23%)	21 (8.24%)	2	0.002*
Anticholinergic drugs (benzhexol) (yes, %)	180 (18.6%)	128 (18%)	52 (20.4%)	2	0.400
Amantadine (yes, %)	77 (8%)	50 (7%)	27 (10.6%)	2	0.080
Dopamine agonists (piribedil) (yes, %)	88 (9.1%)	58 (8.1%)	30 (11.8%)	2	0.099
Dopamine agonists (pramipexole) (yes, %)	544 (56.3%)	394 (55.3%)	150 (58.8%)	2	0.340
MAO-B inhibitors (yes, %)	154 (15.9%)	105 (14.7%)	49 (19.2%)	2	0.110

*Test 1: Student's t-test*.

*Test 2: Chi-square test*.

*Test 3: Mann-Whitney U-test.*

* *Significant difference*.

**Table 2:** Baseline characteristics of motor symptoms of the population.

	**Total** ***n* = 967**	**FOG (-)** ***n* = 712**	**FOG (+)** ***n* = 255**	**Test**	***P*-value**
**Motor symptoms**					
TD score	3.99 ± 3.373	3.90 ± 3.27	4.25 ± 3.63	1	0.172
PIGD score	2.86 ± 1.629	2.60 ± 1.48	3.60 ± 1.79	1	0.000*
Total-PDQ-39	21.21 ± 18.529	17.81 ± 15.43	30.71 ± 22.71	1	0.000*
PDQ-39 dimension-1	4.93 ± 7.543	3.57 ± 5.78	8.75 ± 10.15	1	0.000*
PDQ-39 dimension-2	3.61 ± 4.771	2.69 ± 3.7	6.17 ± 6.27	1	0.000*
PDQ-39 dimension-3	3.83 ± 4.874	3.40 ± 4.39	5.02 ± 5.87	1	0.000*
PDQ-39 dimension-4	2.82 ± 4.405	2.64 ± 4.38	3.34 ± 4.43	1	0.029*
PDQ-39 dimension-5	0.26 ± 0.934	0.23 ± 0.88	0.34 ± 1.07	1	0.171
PDQ-39 dimension-6	3.21 ± 2.877	2.88 ± 2.82	4.16 ± 2.83	1	0.000*
PDQ-39 dimension-7	0.71 ± 1.402	0.69 ± 1.36	0.79 ± 1.51	1	0.303
PDQ-39 dimension-8	1.82 ± 2.076	1.71 ± 1.96	2.14 ± 2.35	1	0.009*
Total-UPDRS	33.76 ± 15.526	31.37 ± 14.31	40.44 ± 16.81	1	0.000*
UPDRS-I	2 ± 1.735	1.91 ± 1.66	2.27 ± 1.91	1	0.007*
UPDRS-II	8.89 ± 4.273	8.25 ± 3.93	10.67 ± 4.68	1	0.000*
UPDRS-III	21.94 ± 11.392	20.45 ± 10.59	26.13 ± 12.49	1	0.000*
PIGD subtype (yes, %)	445 (46%)	317 (44.5%)	128 (50.2%)	2	0.125
TD subtype (yes, %)	461 (47.7%)	357 (50.14%)	104 (40.78%)	2	0.011*
Hoehn-Yahr stage (1,1.5,2,2.5,3)	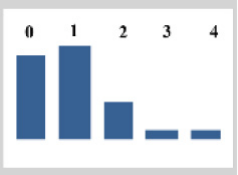	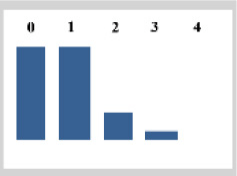	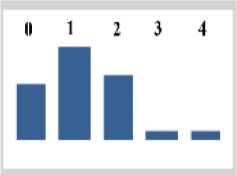	3	0.000*
UPDRS-30 (0, 1, 2, 3, 4)	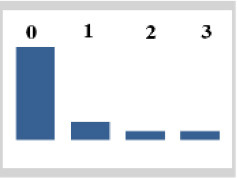	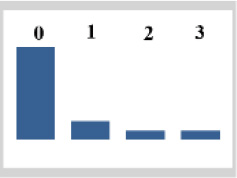	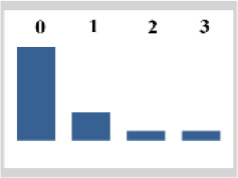	3	0.000*

*Test 1: Student's t-test*.

*Test 2: Chi-square test*.

*Test 3: Mann-Whitney U-test*.

* *Significant difference*.

**Table 3:** Baseline characteristics of non-motor symptoms of the population.

	**Total *n* = 967**	**FOG (-) *n* = 712**	**FOG (+) *n* = 255**	**Test**	***P*-value**
**Non-motor symptoms**					
Total-MMSE	26.97 ± 3.202	27.1 ± 3.13	26.62 ± 3.37	1	0.051
Total-HRS	19.75 ± 6.087	19.97 ± 5.89	19.15 ± 6.57	1	0.079
Total-HDRS17	4.46 ± 4.514	4.05 ± 4.15	5.60 ± 5.25	1	0.000*
Total-PDSS	120.1 ± 26.575	121.62 ± 26.61	115.88 ± 26.08	1	0.003*
Total-PFS	42.72 ± 18.641	40.65 ± 18.1	48.49 ± 18.94	1	0.000*
Total-NMSS	31.06 ± 20.902	28.64 ± 19.36	37.82 ± 23.47	1	0.000*
Hyposmia (yes, %)	400 (41.4%)	286 (40.2%)	114 (44.7%)	2	0.209
Constipation (yes, %)	83 (8.6%)	52 (7.30%)	31 (12.16%)	2	0.026*
EDS (yes, %)	313 (32.4%)	209 (29.35%)	104 (40.78%)	2	0.001*
Total-RBD-HK (yes, %)	328 (33.9%)	220 (30.90%)	108 (42.35%)	2	0.001*
Depression degree	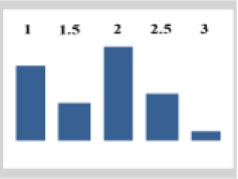	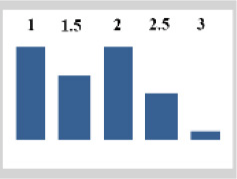	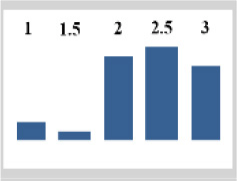	3	0.002*
Depression−0		591 (83.01%)	190 (74.51%)	3	
Depression−1		115 (16.15%)	56 (21.96%)	3	
Depression−2		5 (0.7%)	7 (2.75%)	3	
Depression−3		1 (0.14%)	2 (0.78%)	3	

*Test 1: Student's t-test*.

*Test 2: Chi-square test*.

*Test 3: Mann-Whitney U-test*.

* *Significant difference*.

The patients with PD enrolled had the following features: age at baseline (62.61; SD, 10.01; 95% CI, 61.98–63.24), age of onset (55.68; SD, 10.28; 95% CI, 55.03–56.33), and disease duration at baseline (5.93; SD, 3.63; 95% CI, 5.70–6.16).

Of the 967 patients at the end of the study, 26.4% (255/967) of the patients (119 men and 136 women) were FOG (+) with the following features: age at baseline (64.62; SD, 9.94; 95% CI, 63.40–65.84), age of onset (56.44; SD, 10.44; 95% CI, 55.16–7.72) and disease duration at baseline (7.18; SD, 4.02; 95% CI, 6.69–7.67). Meanwhile, 73.6% (712/967) of the patients (377 men and 335 women) were FOG (–) with the following features: age at baseline (55.41; 10.21; 31–91), age of onset (61.89; 9.94; 8–89), and disease duration at baseline (5.48; 3.37; 1–27).

It was found that the FOG (+) groups had significantly longer disease duration and greater age at baseline than those in the FOG (–) group (*P* < 0.05). There were no significant statistical differences between the FOG (+) and FOG (–) groups in AOO, sex, education level, smoking history, history of alcohol intake, history of exposure to toxic and hazardous substances, history of exposure to heavy metal, history of head injury, surgical history, and family history of PD. There were significantly higher proportions in wearing-off, LEDD, and usage of L-Dopa and COMT inhibitors in patients in the FOG (+) group than those in the FOG (–) group (*P* < 0.05). There were no significant statistical differences found between the FOG (+) and FOG (–) groups in the usage of anticholinergic drugs, amantadine, dopamine agonists, and MAO-B Inhibitors.

The FOG (+) group had a significantly higher Total-UPDRS score, PIGD score, Total-PDQ-39 score, PDQ-39 scores in Dimensions 1, 2, 3, 4, and 6, UPDRS-part I, II, and III scores, and UPDRS-subitem-30 score, and a higher H&Y stage than the FOG (–) group (*P* < 0.05). There were more patients with TD in the FOG (–) group (*P* < 0.05). The FOG (+) group had a significantly higher Total-NMSS score, Total-HDRS-17 score, Total-PFS score, and Total-RBD-HK score than the FOG (–) group (*P* < 0.05), had more patients with constipation, RBD, and EDS than the FOG (–) group (*P* < 0.05) and had more patients that were more likely to have depressive symptoms, and more severely so, than patients with FOG (–) (*P* < 0.05). However, the FOG (+) group had a significantly lower mean score of Total-PDSS than the FOG (–) group (*P* < 0.05). In the parts of MMSE, only parts 1 and IV showed statistical significance between the two groups (*P* < 0.05).

### Risk Factors for FOG and Performance of Prediction Model

All the variables with *P* ≤ 0.1 that were selected by single-factor Cox regression are shown in Supplementary Table 1. The factors possibly linked to FOG were identified by multivariate Cox regression, including the EOPD(±), TD score, Total PFS, PDQ-39 dimension 2, NMSS-29, and UPDRS-30 (see in Table 4). These six risk factors meet the Proportional Hazards (PH) Assumption. The final multivariate Cox regression model was based on the Proportional Hazards ratio Model (1) and was shown below (2). *h* (*t, x*) was named as the prognosis index. β_1_, β_2_ to β_n_ is the partial regression coefficient of independent variables. *h*_0_ (*t*) is the baseline risk when the *x* is 0.


(1) $$\begin{array}{rcl} h\left(t,x\right) & = & h_{0}\left(t\right)\exp\left(\beta_{1}x_{1}+\beta_{2}x_{2}+\ldots +\beta_{\text{p}}x_{\text{p}}\right) \end{array}$$

(2) h(t, x)= h0 (t) exp [(−0.455×EOPD(± ))                   −(0.083×TD Score)+(0.012×Total PFS)                   +(0.06×PDQ.39 Dimension.2)                   +(0.095×NMSS.29)+(0.199×UPDRS.30)]

**Table 4:** Risk factors of FOG selected by multivariate Cox regression.

**Variables**	**B**	**HR (95% CI for HR)**	***P*-value**
EOPD (±)	−0.455	0.634 (0.459–0.876)	0.006
TD-score	−0.083	0.92 (0.883–0.958)	0.000
Total-PFS	0.012	1.012 (1.004–1.02)	0.002
PDQ39-dimension-2	0.06	1.062 (1.036–1.089)	0.000
NMSS-29	0.095	1.1 (1.012–1.196)	0.025
UPDRS-30	0.199	1.22 (1.015–1.467)	0.034

**Figure 2:**
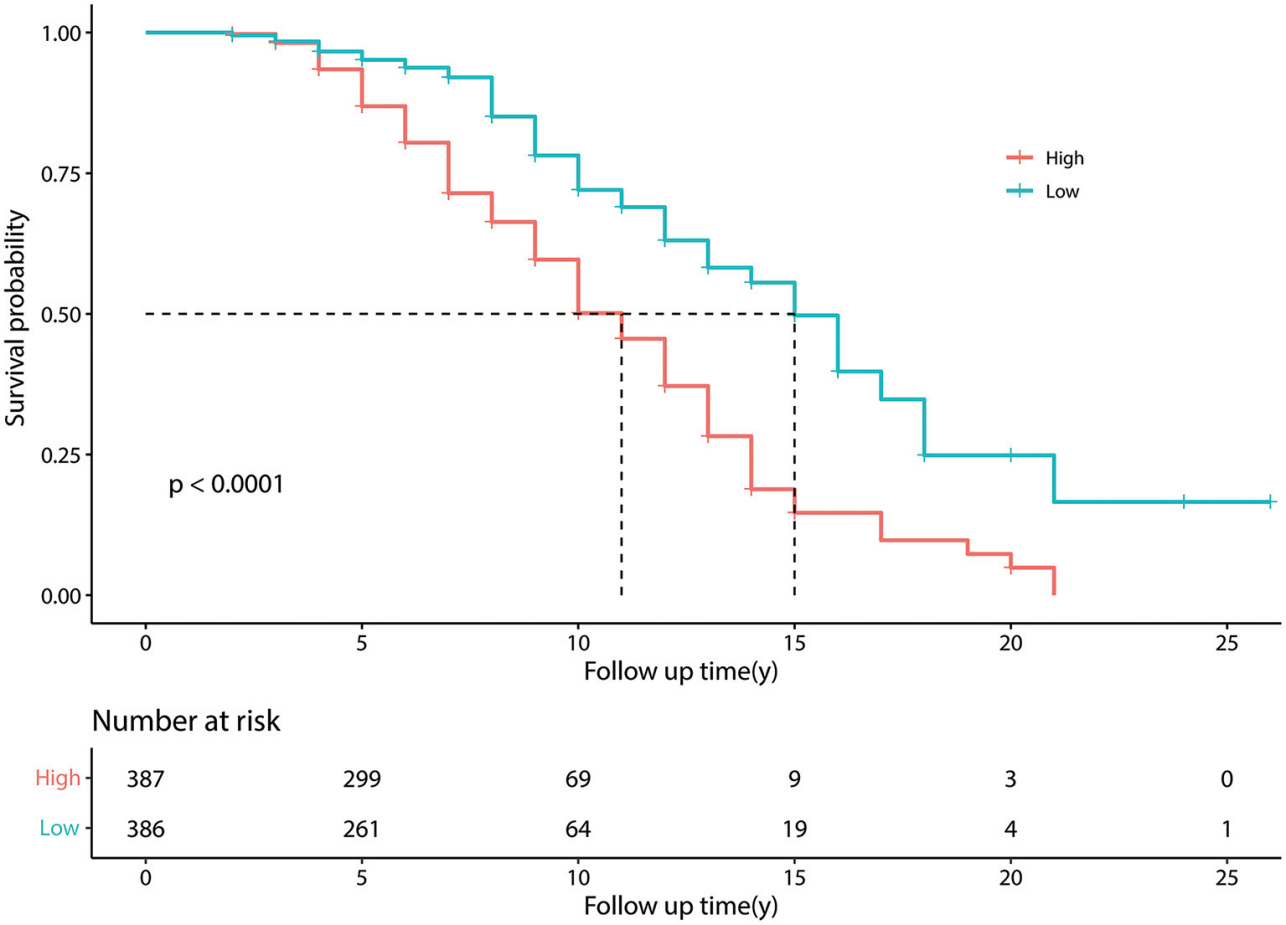
Kaplan–Meier survival curves. Kaplan–Meier curves constructed by the training set, showing that as time went by, there was a significant difference between patients with Parkinson's disease (PD) with a higher risk of developing freezing of gait (FOG) and patients with a lower risk of developing FOG. The number at risk means the number of patients with PD in the group of higher and lower risk of developing FOG; Follow up time (y) means the time from a patient was diagnosed with PD to the end of the follow-up period, in other words means “disease duration at baseline plus 1 year”.

**Figure 3:**
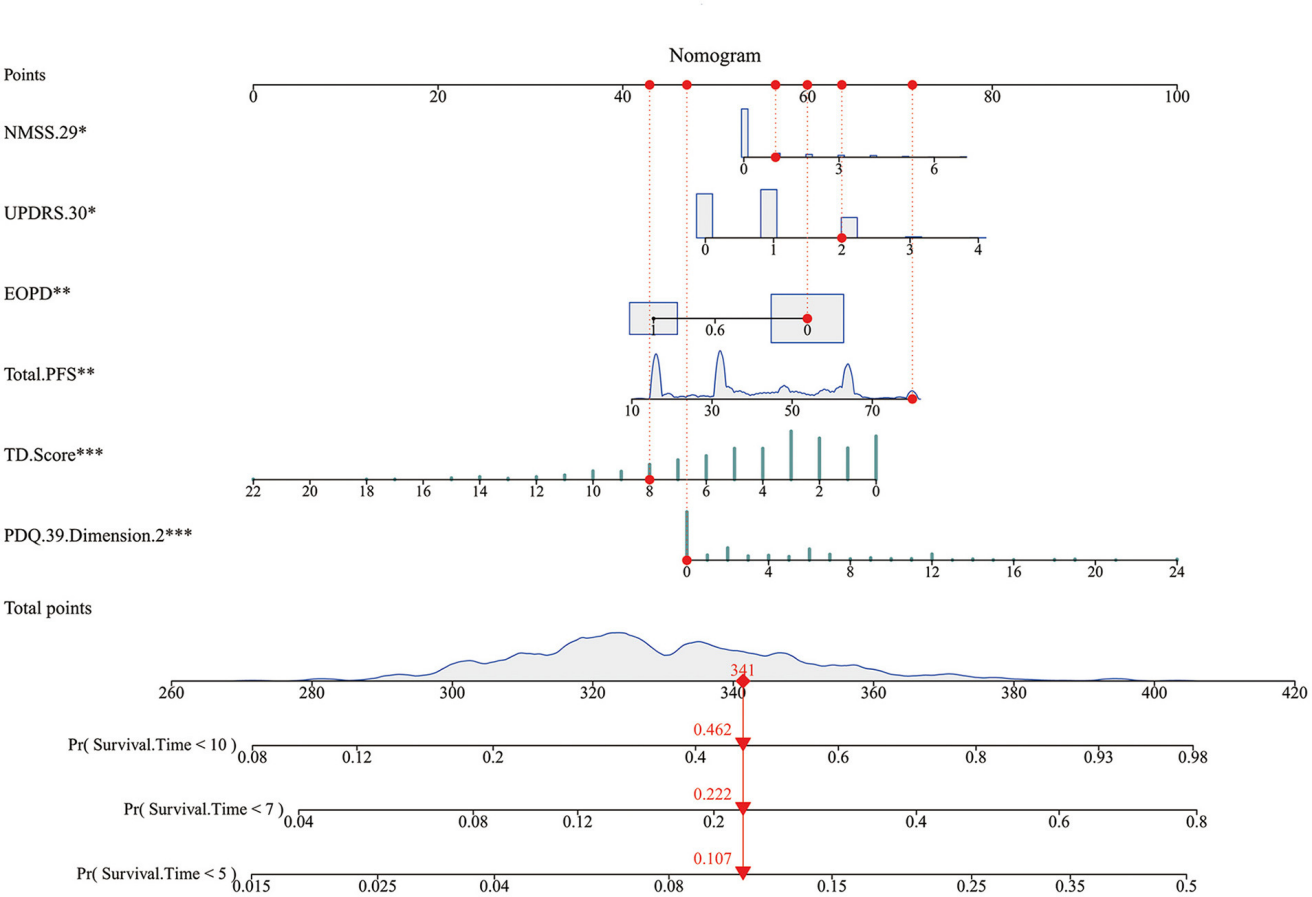
The nomogram. Non-Motor Symptoms Scale (NMSS).29: unexplained weight change; Unified Parkinson's Disease Rate Scale (UPDRS) 0.30: impaired balance; EOPD: early-onset PD; Total. Parkinson's Fatigue Scale (PFS): degree of fatigue; tremor dominant (TD). Score: degree of tremor symptom; Parkinson's Disease Questionnaire (PDQ).39. Dimension.2: Activities of Daily Living. Every factor has a point, and total points are the sum of the point of each factor, corresponding to the probability of FOG occurring at different survival times.

Kaplan-Meier curves showed that, as time went by, there was a significant difference between the patients with PD with a higher risk of developing FOG and the patients with a lower risk of developing FOG (*P* < 0.05) (Figure 2). The nomogram was constructed (Figure 3). The C-indices of the nomogram on the training set and test set were 0.68 and 0.71, respectively. The probability of a PD patient developing FOG after 5, 7, and 10 years of assessment (baseline) can be calculated by the nomogram and validated by the calibration curves.

**Figure 4:**
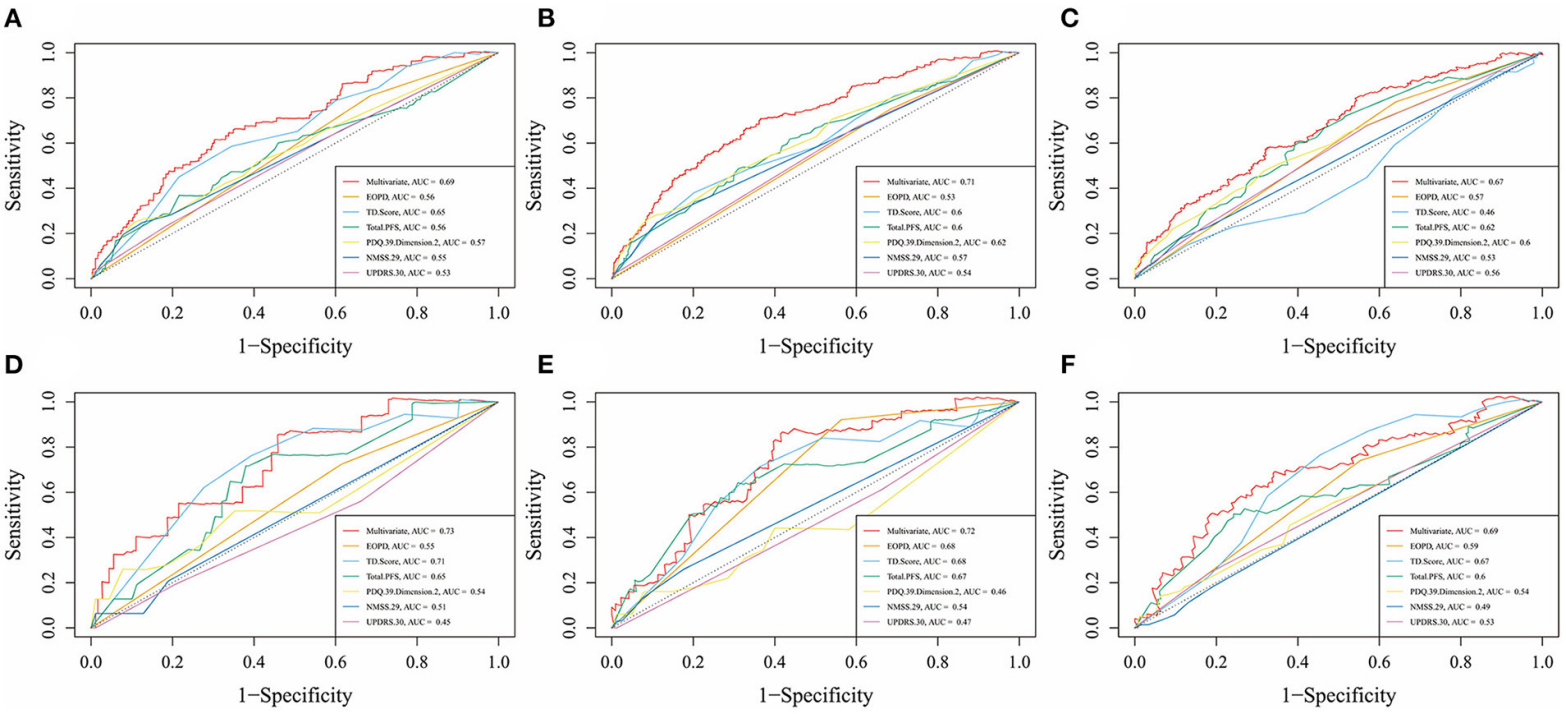
Time-dependent receiver operating characteristic (td-ROC) curves of multivariate and each factor at different survival times in the training set and test set. **(A)** td-ROC of 5-year survival, in the training set; **(B)** td-ROC of 7-year survival, in the training set; **(C)** td-ROC of 10-year survival, in the training set; **(D)** td-ROC of 5-year survival, in the test set; **(E)** td-ROC of 7-year survival, in the test set; **(F)** td-ROC of 10-year survival, in the test set.

**Figure 5:**
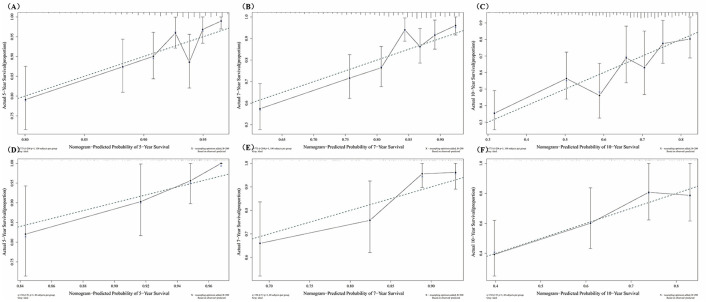
Calibration curves at different survival times in the training set and test set. **(A)** Calibration curves of 5-year survival, in the training set; **(B)** Calibration curves of 7-year survival, in the training set; **(C)** Calibration curves of 10-year survival, in the training set; **(D)** Calibration curves of 5-year survival, in the test set; **(E)** Calibration curves of 7-year survival, in the test set; **(F)** Calibration curves of 10-year survival, in the test set.

Figures 4A–F showed the predictive ability of the multivariate model which was built by the nomogram and every single factor selected by the multivariate Cox regression. It was found that multivariate models had better predictive ability than any other single factors in both training set and test set. In the internal validation set (the training set), the AUC of the multivariate model at 5, 7, and 10 years was 0.69, 0.71, and 0.67, respectively (Figures 4A–C). In the external validation set (the test set), the AUC of the model at 5, 7, and 10 years was 0.73, 0.72, and 0.69, respectively (Figures 4D–F). The calibration curves for 5, 7, 10 years in the internal and external validation sets (training and test sets) were constructed (Figures 5A–F), and the probability of survival at 5 years showed a fair agreement between the prediction by the nomogram and the standard curve in both the training and test sets.

### ML

The training set was used to develop the RF model using ML and the test set was used to validate the RF model. After adjusting the relevant parameters, n_estimators was set to 120, and max_depth was set to 16.

**Figure 6:**
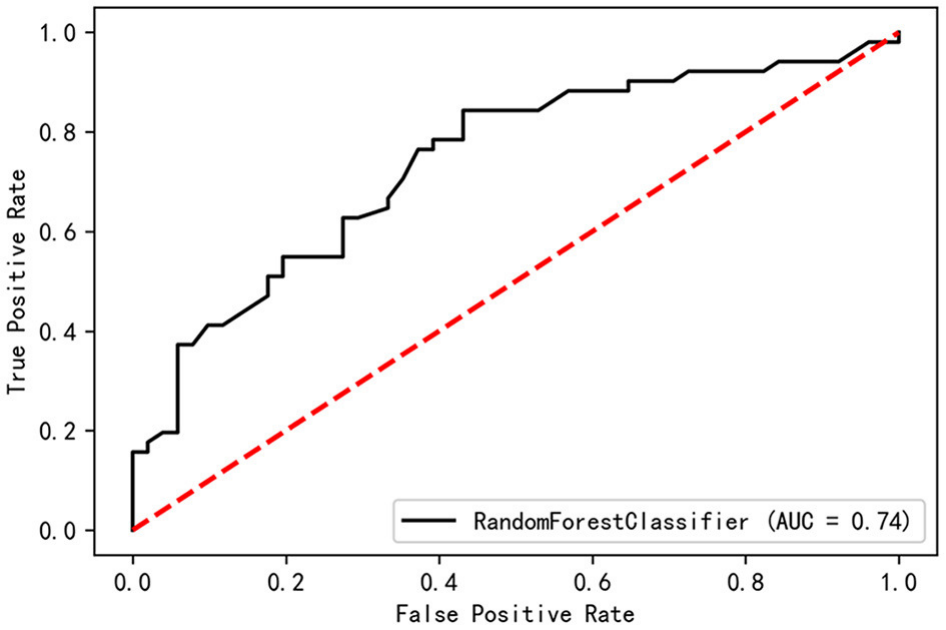
Receiving operating curves (ROC) of Random Forest model.

All the baseline-assessed factors were ranked according to their importance score (Supplementary Table 2). Among the top 30 factors, PDQ-39 Dimension-2, Total-PFS, UPDRS-30, and TD score were present in the nomogram for the Cox regression model. The validation set had a C-index of 0.74 and an AUC of 0.74 (Figure 6).

## Discussion

Gait disorder, especially, FOG is a common motor symptom in PD, and it was reported that gait disorder could be a risk factor or prodromal biomarker of PD (5), indicating that finding out risk factors would be meaningful to clinical physicians. Based on the PD-MDCNC database, PD patients with H&Y stages 1–3 were included in this 1-year longitudinal study. The baseline characteristics of FOG (+) and FOG (–) patients as well as the incidence of FOG in the central-southern region of mainland China were reported, and clinical features statistically linked to FOG development in PD patients were also identified during our study. At the end of the 1-year-long study, 26.4% of the patients suffered from FOG, this was lower than the previous study (3, 35). However, it was still within an acceptable range when we considered the disease duration at baseline of patients we enrolled and the shorter follow-up time in this study. The patients who developed FOG had significantly longer disease duration, greater age at baseline and H&Y stage, lower proportion in TD subtype, a higher proportion in wearing-off, LEDD, usage of L-Dopa and COMT inhibitors, higher scores in scales of UPDRS, PDQ-39, NMSS, HDRS-17, PFS, RBD-HK, ESS, and lower scores in scales of PDSS (*P* < 0.05). These baseline characteristics were coherent with the previous study (15, 35–37).

A nomogram was used to construct the prediction model based on multivariate Cox regression by inputting different clinical PD scales as independent variables. The model had satisfactory prediction efficiencies in the training and test sets at different survival times. The factors associated with the occurrence of FOG included AOO not being under the age of 50 years, a lower degree of tremor symptom, impaired activities of daily living (ADL), UPDRS item 30 posture instability, unexplained weight loss, and a higher degree of fatigue. In addition, according to the ROC curve, any single factor was not as effective as the combination of multiple factors in predicting FOG, indicating that it was a good way to combine with multiple factors related to disease to construct prediction models. RF was used to validate all the variables that might be related to FOG, and the validation set had a C-index of 0.74 and an AUC of 0.74, proving that RF was a good approach to the prediction of FOG. What is more, the importance of risk factors for FOG had been ranked by Gini-index, and most factors found by Cox regression ranked highly, indicating that these factors were important to FOG.

The relationship between FOG and the AOO of PD is unclear. Some studies have demonstrated no significant relationship between the AOO and the development of FOG (15, 36). However, a later AOO is often characterized by the PIGD subtype (38) and the PIGD subtype is a risk factor for the development of FOG (15, 36). Several studies have shown that a later AOO of PD correlates with a faster progression of the disease, especially in imbalance and gait disorders (25). Thus, later AOO may increase the risk of FOG due to a PIGD subtype and faster disease progression.

We used UPDRS sub-items to assess balance and showed that patients with PD exhibiting posture instability have a higher likelihood of developing FOG. The observation that turning exacerbates FOG indicates that an impairment in the ability to control posture is a contributing factor (3). Postural control and balance in patients experiencing FOG were significantly more affected than in non-FOG patients, consistent with the characteristics of the mean PIGD scores in our baseline (39). According to the “threshold model” theory, patients with FOG have more severe gait and balance disorders, FOG occurs when the existing disorders accumulate to a threshold (40). Although there may be a concomitant relationship between impaired balance and FOG, our study still recognizes that impaired balance may be a predictor of FOG.

The PDQ-39 Dimension-2 outlines a variety of ADL conditions crucial for maintaining QoL (26). FOG in PD patients correlates with poor QoL as measured by PDQ-39 (3). Another study found that FOG had a high impact on the QoL of patients with PD (7). Motor functions decrease as PD advances, concomitant with a deterioration in the majority of ADL domains (41), which, in turn, may lead to an increased risk of FOG.

Unexplained weight loss is common in patients with PD and can cause negative health effects (42). A 5-year follow-up study found that weight loss heightened the risk of dependency and dementia, leading to poor prognosis (43), also, dementia was a risk factor of FOG (15). Patients with PD with the PIGD subtype had higher resting metabolic rates and lower body fat, which means patients with the PIGD subtype may more likely to experience weight change (44), and PIGD was identified as a possible risk factor for FOG. What is more, unexplained weight change especially weight loss was correlated with non-dopaminergic symptoms such as gait and balance disorders (45). To sum up, we speculate that unexplained weight change in PD may be associated with an increased risk of FOG.

Fatigue is a disabling and persisting symptom of PD that frequently persists or gets worse with time and negatively impacts the QoL of patients (46). One study revealed the roles of fatigue, as well as PIGD, in the development of FOG (47). A study of the relationship between non-motor symptoms with motor subtypes in PD found that the PIGD subtype was characterized by severe fatigue, which was most negatively impacting the QoL of patients (48). We speculate that fatigue contributes to FOG through the subtype of PIGD and factors that directly affect QoL.

There is less information known about the relationship between the degree of tremor symptom and FOG. Patients with FOG have lower UPDRS part III tremor sub-scores (49), consistent with the baseline characteristics in our study. Another investigation had shown that patients with the TD subtype had a slower disease progression than those with the PIGD subtype, indicating that there is a negative correlation between the TD subtype and the occurrence of FOG (25). This was consistent with the phenomenon that there was a lower proportion in TD subtype in FOG patients in our study, reversely, indicating that a lower degree of tremor symptom might correlate to FOG. Anticholinergics are PD therapies that treat tremor symptoms but do not alleviate akinesia or bradykinesia (50), indicating that there may be a relationship between tremor and cholinergic hyperfunction. PET imaging of patients with PD has found that nigrostriatal and basal forebrain cortical cholinergic denervation is correlated with the slowing of gait in PD (51), and gait disorders may lead to FOG (15, 36). These studies suggested that cholinergic denervation in nigrostriatal and basal forebrain cortical might be associated with FOG and that tremor symptom has a negative correlation with the occurrence of FOG.

Here are the limitations of this study. In this study, the clinical examination and assessment of FOG were mainly based on outpatient consultation and clinical scales assessment. However, due to differences in the educational levels of the patients, it was inevitable that some patients would fail to understand the descriptions of the doctors regarding FOG or fail to recall the detailed progress of their disease. Therefore, some important but mild features might be overlooked in the interview and clinical evaluation, leading to selection bias or recall bias. In this study, the attending physicians and the evaluators repeatedly asked about the symptoms to minimize the impact of these biases. In addition, the period of follow-up (1 year) was relatively short, thus the relatively low incidence of FOG may be related to this. Finally, this study aimed to construct a FOG prediction model suitable for patients with PD of all ages at H&Y stages 1–3, so patients with large age differences were included in the study to cover a wider population. Moreover, due to the large age difference of onset among patients, some discrete values might inevitably exist in the population distribution in this study, and the existence of these values might make the model unable to achieve higher accuracy. This might be the reason why there was no statistically significant difference in the age of onset between the two groups when the baseline characteristics were analyzed. Therefore, in future studies, a better assessment method for FOG and a longer follow-up might be needed.

## Conclusion

This study constructed a 1-year cohort of Chinese patients with PD in the early and middle stages and used a variety of clinical scales to find out risk factors related to FOG, and constructed prediction models by Cox regression and machine learning. In this study, we identified some known risk factors associated with FOG and found that some relatively new factors might be associated with FOG including a lower degree of tremor symptom, unexplained weight loss, and a higher degree of fatigue. The prediction models showed the relatively good ability of prediction, thus providing a simple, convenient way for clinicians to predict FOG in the PD patients with H&Y stages 1–3.

## Data Availability Statement

The original contributions presented in the study are included in the article/Supplementary Material, further inquiries can be directed to the corresponding author/s.

## Ethics Statement

The studies involving human participants were reviewed and approved by the Ethics Committee of Xiangya Hospital of Central South University in China. The patients/participants provided their written informed consent to participate in this study. Written informed consent was obtained from the individual(s) for the publication of any potentially identifiable images or data included in this article.

## Author Contributions

B-sT has full access to all the data in the study, takes responsibility for the integrity of the data and the accuracy of the data analysis, and obtained funding. KX and B-sT contributed to the conception, design of the study, and drafting of the manuscript. KX, X-xZ, R-cH, ZZ, Z-hL, QX, Q-yS, X-xY, J-fG, and B-sT contributed to the acquisition, analysis, or interpretation of data. X-yW, J-fG, and B-sT contributed to administrative, technical, or material support and study supervision. All authors contributed to the critical revision of the manuscript for important intellectual content.

## Funding

This study was funded by the National Key Research and Development Program of China (Grant No. 2016YFC1306000).

## Conflict of Interest

The authors declare that the research was conducted in the absence of any commercial or financial relationships that could be construed as a potential conflict of interest.

## Publisher's Note

All claims expressed in this article are solely those of the authors and do not necessarily represent those of their affiliated organizations, or those of the publisher, the editors and the reviewers. Any product that may be evaluated in this article, or claim that may be made by its manufacturer, is not guaranteed or endorsed by the publisher.

## Abbreviations

ADL: activities of daily living
AOO: age of onset
COMT: catechol-O-methyltransferase
C-index: Concordance index
EDS: excessive daytime sleepiness
EOPD: early onset PD
ESS: Epworth Sleepiness Scale
FOG: freezing of gait
HDRS: Hamilton Depression Rating Scale
Hoehn and Yahr stage: H&Y stage
HRS: Hyposmia Rating Scale
LEDD: levodopa equivalent daily dosage
ML: machine learning
MMSE: mini-mental state examination
NFOG-Q: New Freezing of Gait-Questionnaire
NMSS: Non-Motor Symptoms Scale
PD: Parkinson's Disease
PDQ-39: 39-item Parkinson's Disease Questionnaire
PFS: Parkinson's Fatigue Scale
PIGD: postural instability gait difficulty
QoL: quality of life
RBDQ-HK: rapid eye movement sleep behavior disorder questionnaire-Hong Kong
RF: random forest
TD: tremor dominant
UPDRS: Unified Parkinson's Disease Rate Scale.
