# Supplementary material for: Constructing Prediction Models for Freezing of Gait by Nomogram and Machine Learning: A Longitudinal Study
Source: Front Neurol. 2021 Dec 6;12:684044. doi: 10.3389/fneur.2021.684044 (PMC8686836; doi:10.3389/fneur.2021.684044)
Supplement: Supplementary file 1 [file Table_1.DOCX]

**Supplementary Table 1**

Risk factors of FOG selected by Single-factor Cox regression.

| **Variables** | **Beta** | **HR (95% CI for HR)** | **P-value** |
| --- | --- | --- | --- |
| EOPD(±) | -0.57 | 0.57 (0.41-0.78) | 0.00048** |
| Family History of PD | -0.55 | 0.58 (0.35-0.97) | 0.037** |
| Sex | -0.31 | 0.74 (0.56-0.97) | 0.03** |
| Education Level | -0.35 | 0.71 (0.47-1.1) | 0.1* |
| TD Score | -0.039 | 0.96 (0.93-1) | 0.043** |
| PIGD Score | 0.13 | 1.1 (1.1-1.2) | 0.001** |
| MMSE-Ⅳ | -0.12 | 0.89 (0.77-1) | 0.091* |
| Total HDRS17 | 0.024 | 1 (1-1) | 0.027** |
| Depression Degree | 0.22 | 1.2 (1-1.5) | 0.051* |
| Hoehn-Yahr Stage | 0.13 | 1.1 (1-1.3) | 0.022** |
| Total PFS | 0.015 | 1 (1-1) | 0.00013** |
| Amantadine(±) | -0.4 | 0.67 (0.44-1) | 0.075* |
| Single Dose of Amantadine | -0.0037 | 1 (0.99-1) | 0.1* |
| Total Daily Dose of Amantadine | -0.0018 | 1 (1-1) | 0.083* |
| Total PDQ-39 | 0.015 | 1 (1-1) | 2.50E-06** |
| PDQ-39 Dimension-1 | 0.031 | 1 (1-1) | 9.00E-06** |
| PDQ-39 Dimension-2 | 0.064 | 1.1 (1-1.1) | 8.00E-08** |
| PDQ-39 Dimension-3 | 0.037 | 1 (1-1.1) | 0.005** |
| PDQ-39 Dimension-5 | 0.12 | 1.1 (0.99-1.3) | 0.069* |
| PDQ-39 Dimension-6 | 0.04 | 1 (0.99-1.1) | 0.083* |
| Total NMSS | 0.0096 | 1 (1-1) | 0.0021** |
| NMSS-1 | 0.11 | 1.1 (1-1.2) | 0.023** |
| NMSS-4 | 0.084 | 1.1 (1-1.2) | 0.0042** |
| NMSS-9 | 0.063 | 1.1 (0.99-1.1) | 0.074* |
| NMSS-10 | 0.078 | 1.1 (1-1.2) | 0.03** |
| NMSS-12 | 0.097 | 1.1 (1-1.2) | 0.057* |
| NMSS-13 | 0.13 | 1.1 (0.99-1.3) | 0.06* |
| NMSS-14 | 0.16 | 1.2 (1-1.4) | 0.039** |
| NMSS-22 | 0.057 | 1.1 (0.99-1.1) | 0.08* |
| NMSS-24 | 0.084 | 1.1 (1-1.1) | 0.0023** |
| NMSS-27 | 0.056 | 1.1 (0.99-1.1) | 0.081* |
| NMSS-29 | 0.13 | 1.1 (1-1.2) | 0.0029** |
| UPDRS-3 | 0.14 | 1.1 (0.99-1.3) | 0.072* |
| UPDRS-11 | 0.19 | 1.2 (0.96-1.5) | 0.1* |
| UPDRS-12 | 0.21 | 1.2 (0.98-1.6) | 0.072* |
| UPDRS-15 | 0.23 | 1.3 (1-1.6) | 0.043** |
| UPDRS-16 | -0.18 | 0.83 (0.7-0.98) | 0.032** |
| UPDRS-17 | 0.18 | 1.2 (1-1.4) | 0.017** |
| UPDRS-20A | -0.23 | 0.79 (0.61-1) | 0.091* |
| UPDRS-20B | -0.23 | 0.79 (0.6-1) | 0.097* |
| UPDRS-20C | -0.17 | 0.84 (0.71-0.99) | 0.037** |
| UPDRS-22B | 0.13 | 1.1 (0.98-1.3) | 0.092* |
| UPDRS-22C | 0.12 | 1.1 (0.99-1.3) | 0.08* |
| UPDRS-26A | 0.14 | 1.2 (0.99-1.3) | 0.059* |
| UPDRS-26B | 0.16 | 1.2 (1-1.4) | 0.027** |
| UPDRS-27 | 0.28 | 1.3 (1.1-1.6) | 0.005** |
| UPDRS-28 | 0.23 | 1.3 (1.1-1.5) | 0.011** |
| UPDRS-29 | 0.21 | 1.2 (0.99-1.5) | 0.057* |
| UPDRS-30 | 0.31 | 1.4 (1.1-1.6) | 0.00055** |
| UPDRS-31 | 0.19 | 1.2 (1-1.5) | 0.043** |
| TD Subtype | -0.3 | 0.74 (0.56-0.98) | 0.034** |
| *Significant difference(0.1); ** Significant difference(0.05) | | | |

**Supplementary Table 2**

All factors importance rank of FOG selected by RF.

| **Feature** | **Importance** |
| --- | --- |
| PDQ-39 Dimension-2 | 0.034633258 |
| Total-PDQ-39 | 0.034536509 |
| Total-UPDRS | 0.031444146 |
| PDQ-39 Dimension-1 | 0.029217331 |
| UPDRS-Ⅲ | 0.02667158 |
| UPDRS-Ⅱ | 0.025677423 |
| Total-PFS | 0.025650902 |
| Survival Time | 0.024805052 |
| Total-NMSS | 0.022542634 |
| Total-PDSS | 0.021150346 |
| LEDD | 0.020590242 |
| Hoehn-Yahr Stage | 0.019511915 |
| PDQ-39 Dimension-6 | 0.018121007 |
| PIGD Score | 0.01615572 |
| NMSS-4 | 0.015372534 |
| TD Score | 0.01393327 |
| PDQ-39 Dimension-3 | 0.013659286 |
| Total-HDRS17 | 0.013478424 |
| NMSS-24 | 0.012123471 |
| Total-MMSE | 0.011539584 |
| UPDRS-26B | 0.010251987 |
| NMSS-27 | 0.009915676 |
| UPDRS-Ⅰ | 0.009739768 |
| NMSS-22 | 0.00964497 |
| UPDRS-17 | 0.008979258 |
| UPDRS-10 | 0.008936982 |
| NMSS-5 | 0.00892765 |
| NMSS-3 | 0.008786079 |
| UPDRS-30 | 0.008596538 |
| PDQ-39 Dimension-4 | 0.008422519 |
| Total-HRS | 0.008318409 |
| UPDRS-26A | 0.008024757 |
| NMSS-10 | 0.007801974 |
| Single Dose of L-dopa | 0.007651116 |
| NMSS-19 | 0.007597797 |
| NMSS-21 | 0.007551528 |
| Age | 0.007547987 |
| Total Daily Dose of Levodopa | 0.007522267 |
| LED of Single Dose of DA | 0.007482901 |
| UPDRS-22E | 0.007464255 |
| NMSS-28 | 0.007292973 |
| NMSS-9 | 0.007147473 |
| UPDRS-28 | 0.007042386 |
| UPDRS-22C | 0.007002381 |
| LED of Total Daily Dose of DA | 0.006971019 |
| NMSS-17 | 0.006965865 |
| PDQ-39 Dimension-7 | 0.00696041 |
| PDQ-39 Dimension-8 | 0.006957385 |
| MMSE-Ⅲ | 0.006825769 |
| UPDRS-31 | 0.006779544 |
| UPDRS-16 | 0.006564242 |
| UPDRS-22B | 0.006355868 |
| Sex | 0.006339562 |
| UPDRS-25B | 0.006256044 |
| NMSS-30 | 0.006230649 |
| MMSE-Ⅳ | 0.006109886 |
| UPDRS-23B | 0.006073214 |
| UPDRS-15 | 0.005888031 |
| NMSS-18 | 0.005837033 |
| NMSS-6 | 0.005711532 |
| MMSE-Ⅴ | 0.005707267 |
| UPDRS-6 | 0.005690067 |
| NMSS-23 | 0.005634715 |
| NMSS-20 | 0.005450221 |
| NMSS-1 | 0.005431478 |
| UPDRS-22D | 0.005291948 |
| UPDRS-23A | 0.005247671 |
| NMSS-11 | 0.00517171 |
| Total-RBD-HK | 0.005124177 |
| UPDRS-24B | 0.005119594 |
| UPDRS-19 | 0.005103576 |
| UPDRS-20C | 0.005087645 |
| UPDRS-21B | 0.005024822 |
| UPDRS-29 | 0.005017998 |
| UPDRS-8 | 0.004931913 |
| UPDRS-25A | 0.004891235 |
| UPDRS-20E | 0.004863105 |
| NMSS-8 | 0.004803815 |
| NMSS-12 | 0.004771267 |
| UPDRS-24A | 0.004696385 |
| Total Daily Dose of Anticholinergic Drugs | 0.00463153 |
| NMSS-7 | 0.004542018 |
| UPDRS-11 | 0.004529246 |
| NMSS-15 | 0.004455185 |
| UPDRS-3 | 0.004371785 |
| UPDRS-9 | 0.004367077 |
| UPDRS-4 | 0.004259843 |
| UPDRS-22A | 0.004227503 |
| NMSS-16 | 0.004200702 |
| UPDRS-1 | 0.004189603 |
| UPDRS-18 | 0.004006028 |
| UPDRS-2 | 0.003985438 |
| UPDRS-12 | 0.003953035 |
| LED of Single Dose of MAOB Inhibitors | 0.003827438 |
| UPDRS-5 | 0.003781795 |
| UPDRS-21A | 0.003690783 |
| UPDRS-20D | 0.003547173 |
| MMSE-I | 0.003464595 |
| UPDRS-20A | 0.003407302 |
| UPDRS-27 | 0.003375319 |
| TD Subtype | 0.003326552 |
| EDS | 0.003311946 |
| NMSS-29 | 0.003304364 |
| EOPD | 0.003226612 |
| PIGD Subtype | 0.003212151 |
| LED of Total Daily Dose of MAOB Inhibitors | 0.003178325 |
| MAO-B Inhibitors（±） | 0.003171139 |
| Smoking History | 0.003068886 |
| Single Dose of Anticholinergic Drugs | 0.002961657 |
| Family History of PD | 0.002960384 |
| Education Level | 0.00284119 |
| UPDRS-7 | 0.002746678 |
| Operation History | 0.002729225 |
| Wearing-off? | 0.002663048 |
| PDQ-39 Dimension-5 | 0.002658945 |
| Hyposmia | 0.002644591 |
| NMSS-13 | 0.002524889 |
| Total Daily Dose of Amantadine | 0.002516676 |
| Anticholinergic Drugs（Benzhexol）（±） | 0.002369669 |
| Depression Degree | 0.002350682 |
| UPDRS-13 | 0.002085975 |
| MMSE-Ⅱ | 0.001943715 |
| Amantadine（±） | 0.001925092 |
| Constipation | 0.001879287 |
| Dopamine Agonists（Pramipexole） | 0.001848283 |
| Single Dose of Amantadine | 0.001747722 |
| History of Alcohol Intake | 0.001739134 |
| Single Dose of COMT Inhibitors | 0.001598499 |
| NMSS-2 | 0.001532077 |
| UPDRS-20B | 0.001430971 |
| History of Exposure to Heavy Metal Contamination | 0.001421849 |
| NMSS-14 | 0.001334228 |
| Total Daily Dose of COMT Inhibitors | 0.001307858 |
| Dopamine Agonists（Piribedil） | 0.001297007 |
| Levo-Dopa（±） | 0.001184243 |
| History of Exposure to Pesticides | 0.001163843 |
| Head Injury | 0.000820843 |
| COMT Inhibitors（±） | 0.000763598 |
| History of Exposure to Organic Solvent | 4.38E-05 |
